# miR-30b-5p Downregulation as a Predictive Biomarker of Coronary In-Stent Restenosis

**DOI:** 10.3390/biomedicines9040354

**Published:** 2021-03-30

**Authors:** Encarnación Gutierrez-Carretero, Isabel Mayoral-González, Francisco Jesús Morón, Mónica Fernández-Quero, Alejandro Domínguez-Rodríguez, Antonio Ordóñez, Tarik Smani

**Affiliations:** 1 Cardiovascular Pathophysiology, Institute of Biomedicine of Seville, University Hospital of Virgen del Rocío, University of Seville, CSIC, 41013 Seville, Spain; gutierrez.encarnita@gmail.com (E.G.-C.); isabelmayoralgon@hotmail.com (I.M.-G.); aleprody@hotmail.com (A.D.-R.); antorfernan@us.es (A.O.); 2 Department of Surgery, Faculty of Medicine, University of Seville, 41009 Sevilla, Spain; 3 University Hospital Virgen del Rocío, 41013 Sevilla, Spain; fdezqueromonica@yahoo.es; 4 Genomic Facility, Institute of Biomedicine of Seville, University Hospital of Virgen del Rocío, University of Seville, CSIC, 41013 Seville, Spain; mcivanto-ibis@us.es; 5 Department of Medical Physiology and Biophysics, Faculty of Medicine, University of Seville, 41009 Seville, Spain

**Keywords:** Primary Coronary Intervention, in-stent restenosis, miRNAs, biomarker

## Abstract

In-stent restenosis (ISR) is one of the main limitations of percutaneous coronary intervention (PCI) therapy with drug-eluting stents (DES) implantation. The aim of this study was to determine if circulating microRNAs (miRNAs) have diagnostic capability for determining ISR in a cohort of matched patients. Blood samples were collected from 55 patients who underwent previously PCI and were readmitted for a new coronary angiography. Patients were divided into subgroups comprising patients who presented ISR or not (non-ISR). A microarray analysis determined that up to 49 miRNAs were differentially expressed between ISR and non-ISR patients. Of these, 10 miRNAs are related to vascular smooth muscle and endothelial cells proliferation, migration, and differentiation, well-known hallmarks of vascular remodeling. Additionally, we identified that the expression of miR-30b-5p is significantly lower in serum samples of ISR patients, as compared to non-ISR. A further analysis demonstrated that miR-30b-5p provides better values of the receiver operator characteristic curve than other miRNAs and biochemical parameters. Finally, the in-silico analysis suggests that miR-30b-5p is predicted to target 62 genes involved in different signaling pathways involved in vascular remodeling. In conclusion, we determined for the first time that circulating mi-R30b-5p can reliably prognose restenosis in patient with implanted DES, which could be potentially helpful in the establishment of an early diagnosis and therapy of ISR.

## 1. Introduction

Coronary artery disease (CAD) is considered the major cause of death and disability in developed countries [1]. CAD is mainly associated with atherosclerosis plaque deposition in the intima of coronary arteries, where it narrows the artery diameters, which limits or even blocks the blood flow [2]. The atherosclerotic progress is silent until the appearance of clinically manifestations as angina pectoris, acute myocardial infarction (AMI), or even sudden coronary death [3]. Percutaneous coronary intervention (PCI) has significantly improved the prognosis for patients with CAD, reducing clinical symptoms. However, restenosis, defined as the repeated narrowing of the vessel diameter, remains a serious limitation of this procedure [4]. The main cause of restenosis was attributed to the excessive proliferation and migration of vascular smooth muscle cells (VSMC) to the intima [5] and their switch from contractile to synthetic phenotypes. Nowadays, drug-eluting stents (DES), mainly targeting VSMC proliferation and migration, are widely used, which drastically reduces the rate and the incidence of in-stent restenosis (IRS). Actually, the use of DES achieved significant relief of clinical symptoms and reduced CAD patients mortality, as reviewed recently [6]. Currently, conventional and computed coronary tomography angiographies are the only effective strategies of detecting ISR in patients presenting AMI-like symptoms. However, the consequences of successive coronary angiography: its high cost, its invasive character, and its test contradictions make urgent the need of a sensitive and reliable marker to diagnose restenosis, which could influence positively a preventive treatment, especially in high-risk patients.

MicroRNAs (miRNAs) are small noncoding RNAs with approximately 19–25 nucleotides involved in regulating a wide range of developmental and physiological processes [7]. MiRNAs play critical role in the regulation of gene expression at the post-transcriptional level [8] and in the modulation of the cellular mechanism implicated in cardiovascular diseases (CVDs), such as cardiac hypertrophy, cardiac arrhythmias, or heart failure [9]. MiRNAs could be released after cellular activation, stress, or injury [10], so they are considered as valuable and stable biomarkers for heart disease, such as restenosis, as reviewed elsewhere [2]. However, little is known regarding the expression of miRNAs in patients who underwent PCI and developed, or not, in-stent restenosis (ISR). In the present study, we evaluated the diagnostic performance of circulating miRNAs as serum biomarkers for patient who have developed ISR.

## 2. Experimental Section

**Figure 1:**
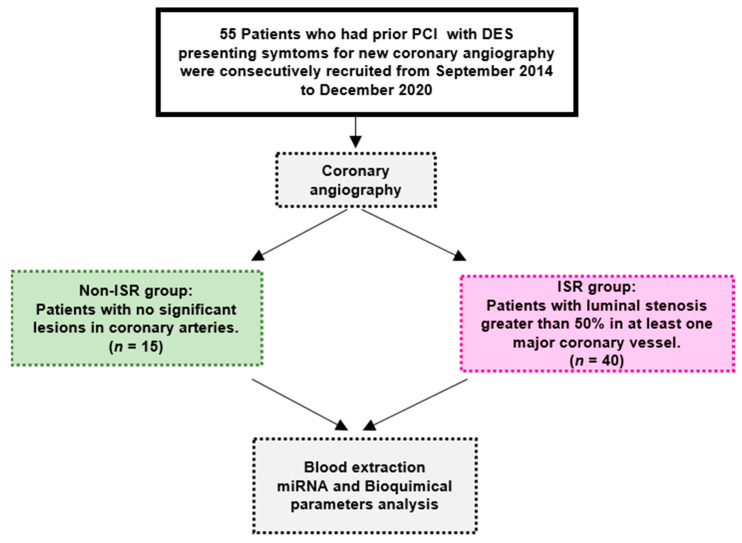
Strengthening the reporting of observational studies in epidemiology (STROBE) diagram of the subjects and the study flow. DES: drug-eluting stent, ISR: patient with in-stent restenosis, non-ISR: patient with non in-stent restenosis, miRNA: microRNA, and PCI: percutaneous coronary interventions.

This single-center study was conducted according to the Ethical Principles of the Declaration of Helsinki. It was approved by the Ethics Committee on Human Research at the University Hospital “Virgen del Rocio” of Seville (approval no. pi-0313-2016; date of approval: 2 February 2017). The Strengthening the reporting of observational studies in epidemiology (STROBE) guidelines were followed to report our findings (Figure 1).

### 2.1. Study Subjects and Data Collection

This retrospective study was conducted in 55 patients undergoing coronary angiography who had prior coronary angiography with DES implantation and met the eligibility criteria based on symptoms suggesting ischemia, such as chest pain radiation to arm, neck, or jaw. In-stent restenosis (ISR) and non-ISR groups were derived from patients undergoing coronary angiography. ISR was defined as the luminal stenosis greater than 50% in at least one major coronary vessel. Patients with allergy to iodine or with an acute renal failure were excluded from this study. Patients provided informed consent and were enrolled in consecutive series in this study.

Coronary artery lesions characteristics were analyzed after coronary angiography, such as multivessel artery lesions and target lesion at the left anterior descending (LAD) artery, at the left circumflex (LCX) artery, and at the right coronary artery (RCA). Baseline, postoperative, and follow-up coronary angiograms were digitally recorded, and the quantitative coronary angiography (QCA) analyses were performed with an automated edge-detection system Carestream (Medis Medical Imaging Systems, Leiden, The Netherland). We also collected and analyzed clinical data, such as age, gender, smoking conditions, hypertension, diabetes mellitus (DM), insulin therapy, chronic kidney disease (CKD), hemoglobin, creatinine, creatine phosphokinase (CPK), C-reactive protein (CRP), troponin, N-terminal natriuretic peptide (NT-proBNP), total cholesterol (TC), high-density lipoprotein cholesterol (HDL-C), low-density lipoprotein cholesterol (LDL-C), triglycerides (TG), erythrocytes, leucocytes, and neutrophils.

### 2.2. Blood Extraction, miRNA Isolation, and Quantification

Blood samples were collected from patients before the second angiography motivated by the recurrence of ischemic symptoms. Blood samples were centrifuged at 3000 rpm for 15 min to separate the serum that were later processed in the laboratory for RNA extraction using the miRNeasy Serum/Plasma kit (Qiagen, Hilden, Germany) to extract small RNAs. Two hundred microliters of serum was mixed with 700 μL of QIAzol^®^ lysis reagent included in the kit. RNA quantity was determined using fluorometric quantification by the Qubit miRNA assay (Thermo Fisher Scientific, Waltham, MA, USA).

### 2.3. GeneChip miRNA Arrays

The total RNA was labeled using the FlashTag^®^ Biotin HSR labeling Kit (Thermo Fisher Scientific, Inc., Waltham, MA, USA) following the manufacturer’s indications. We used GeneChip^®^ miRNA 4.0 arrays (Thermo Fisher Scientific, Inc., Waltham, MA, USA) to analyze the expression of miRNAs. Washing, staining (GeneChip^®^ Fluidics Station 450), and scanning (GeneChip^®^ Scanner 3000, Thermo Fisher Scientific, Inc., Waltham, MA, USA) were performed following the protocols outlined in the user manual for cartridge arrays. Briefly, CEL file import and miRNA level with robust multiarray average (RMA) normalization and detection above the background (DABG) were performed using Transcriptome Analysis Console (TAC) 4.0 software (Thermo Fisher Scientific, Inc., Waltham, MA, USA). The miRNA 4.0 arrays contain 6631 human small noncoding RNA transcripts involved in gene regulation. A comparative analysis between samples of ISR and non-ISR patients was carried out using a fold change of over ±1.3 and a univariate ANOVA analysis with a *p*-value < 0.05, in which the null hypothesis was “there is no difference between the groups”. In addition, a probe set was considered expressed if ≥ 50% samples had DABG values below the threshold (DABG < 0.05). Hierarchical clustering was performed using complete linkage and Euclidean distance as a measure of similarity for the differentially expressed with TAC 4.0 software.

### 2.4. miRNA Quantification by qRT-PCR

One microgram of small RNAs were reverse-transcribed into cDNA using the miScript II RT Kit (Qiagen, Hilden, Germany), and the cDNA product was diluted up to 1:10. qRT-PCR was performed using an Applied Biosystems Viia7 7900HT thermocycler and FrameStar 384-Well PCR Plate (4titude, Wotton, UK) designed to detect the expression of three miRNAs of interest and two endogenous controls in 14 samples per plate. PCR mix included 10 × universal primer (included in the miScript SYBR Green PCR Kit from Qiagen (Hilden, Germany)). SYBR Green reactive (iTaq™ Universal SYBR Green Supermix, Bio-Rad, CA, USA) 10× universal primer (miScript SYBR Green PCR Kit, Qiagen, Hilden, Germany), specific oligos for each miRNA (miScript Primer Ctrl_miRTC_1, miScript Primer Hs_miR_339-5p, miScript Primer Hs_miR-3916_1, and miScript Primer Hs_miR_30b, all from Qiagen, Hilden, Germany) and cDNA. Thermal cycling conditions were as follows: 95 °C for 20 s, followed by 45 cycles of 95 °C for 1 s and 60 °C for 20 s. Analysis was accomplished with Quant Studio Real-Time PCR software, and data were calculated. The log-fold change in a logarithmic scale was used for the comparative cycle threshold CT (ΔΔCT) method, using RTC-1 as the endogenous control. To identify the miRNA target gene pathways, we used an online platform from the Gene Ontology (GO) browser PANTHER (Protein Analysis THrough Evolutionary Relationships, 14.1 version http://pantherdb.org/ (accessed on 26 March 2021).).

### 2.5. Statistical Analysis

Data were analyzed using GraphPad (GraphPad Software, Inc., San Diego, CA, USA). The results are presented as the mean and standard error of the mean (S.E.M). The outliers were removed based on the results of QuickCalcs, an online tool of GraphPad. The Shapiro–Wilk test was used for normality. For normally distributed variables, we used the *t*-test without correction (Fisher’s least significant difference (LSD) test). Linear regression analysis was conducted using the biochemical parameters and miRNA fold change (FC). The predictive values of the miRNAs were also evaluated using the receiver operating characteristic (ROC) curve.

## 3. Results

### 3.1. Clinical Characteristics of the Patients

As outlined in Figure 1 and Table 1, 55 patients enrolled in this study were divided into two group based on the angiography results. The median age of the cohort was 65.31 ± 1.51 years, and 44 patients (80%) were male. During the first PCI, 96 DES were implanted in different coronary arteries. Fifty percent of patients had only one stent, 15% two stents, and 35% more than three stents implanted. DES (68.8%) were placed in the LAD, 20.8 % in LCX, and 10.4% in RCA. The second coronary angiography was indicated according to the clinical guidelines due to angina pectoris in 60.1% of the patients, acute coronary symptoms (ACS) in 34.5%, a kidney pretransplantation in 1.8% patients, and to heart failure in 3.6%. The angiography analysis indicated that 72.7% of these patients had luminal stenosis greater than 50% in at least one major coronary vessel, who underwent a second PCI with DES implantation. The mean duration from stent implantation to the diagnosis of ISR or non-ISR by coronary angiography was 3.5 ± 0.7 years. During the second PCI, 58 DES were implanted in coronary arteries. DES (53.4%) were placed in the LAD, 31.1 % in the LCX, and 15.5% in the RCA.

**Figure 2:**
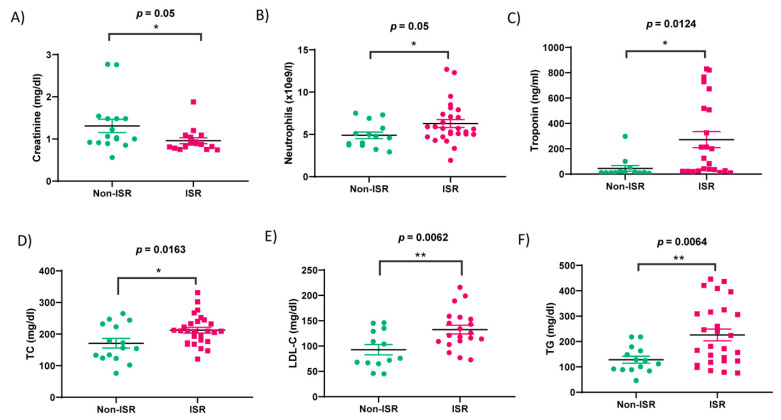
Hemodynamic parameters of the patient groups. Green and rose colors indicate non-ISR and ISR patients, respectively. Bar graphs show (**A**) the concentration of creatine, (**B**) neutrophils, (**C**) Troponin, (**D**) total cholesterol (TC), (**E**) Low-density Lipoprotein Cholesterol (LDL-C), and (**F**) triglycerides (TG) assessed in the serum sample collected from non-ISR and ISR patients. Values are the means ± S.E.M. “*” and “**” indicate significance at *p* < 0.05 and *p* < 0.001, respectively.

**Figure 3:**
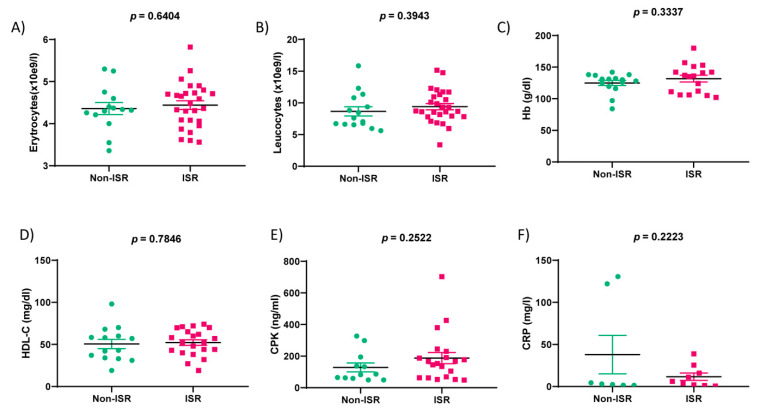
Hemodynamic parameters of the non-ISR (green) and ISR (rose) patients’ groups. Bar graphs show the concentration/levels of erythrocytes (**A**), leukocytes (**B**), hemoglobin (Hb) (**C**), High-Density Lipoprotein cholesterol (HDL-C) (**D**), Creatine phosphokinase (CPK) (**E**), C-Reactive Protein (CRP) (**F**) assessed in the serum sample collected from the non-ISR and ISR patients. Values are the means ± S.E.M.

As detailed in Table 1, the non-ISR group was not significantly different from the ISR group in terms of age, gender, smoking status, diabetes, or dyslipidemia. Although, the number of DES implanted in RCA was higher than those in non-ISR patients. The second PCI showed that stenosis again was concentrated in LAD, followed by LCX and RCA. In addition, as analyzed in Figure 2 and Table 2, we observed significant differences in the six parameters: neutrophils (*p*-value = 0.05), creatinine (*p*-value = 0.05), troponin (*p*-value = 0.0124), total cholesterol (TC; *p*-value = 0.0163), low-density lipoprotein cholesterol (LDL-C; *p*-value = 0.0062), and triglycerides (TG; *p*-value = 0.0064). Meanwhile, no significant changes were observed in the levels of erythrocytes, leucocytes, hemoglobin (Hb), high-density lipoprotein cholesterol (HDL-C), creatine phosphokinase (CPK), and C-reactive protein (CRP) (Figure 3).

### 3.2. miRNA Expression Profiles in ISR Patients

**Figure 4:**
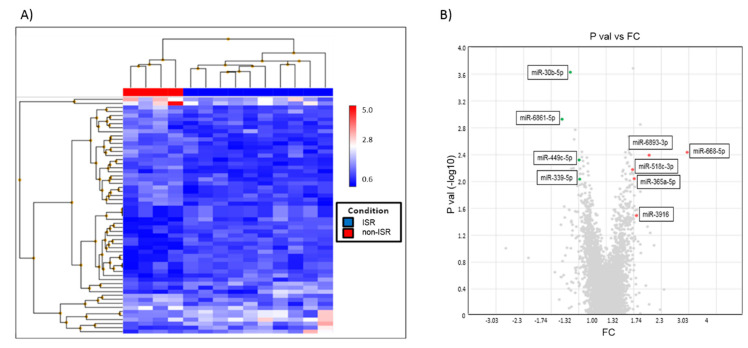
Microarray analysis of the expression of miRNAs in ISR as compare to non-ISR patients. (**A**) Hierarchical clustered sample-centric heat-map analysis of the ΔCt value of differentially expressed miRNAs in ISR (red) and non-ISR patients (blue). Distance was measured by Pearson’s correlation. (**B**) Volcano Plot showing differentially expressed miRNAs between non-ISR and ISR are represented by splashes which show fold-change (FC) values and *p* values of miRNAs. Green splashes indicate significantly upregulated miRNAs. Red splashes indicate significantly downregulated miRNAs.

Serum samples from ISR and non-ISR patients were used to detect the profiles of circulating miRNAs by the qRT-PCR-based array. The analysis of the hierarchical clustering (Figure 4A) and volcano plot (Figure 4B) indicates significant alterations in the expression of 49 miRNAs (fold change ± 1.3 and *p* < 0.05); 31 miRNAs were upregulated and 18 downregulated. Next, we selected 10 miRNAs with the maximum fold change and significant differences between the patient groups to analyze their implications in the signaling pathway, focusing on those relevant for smooth muscle proliferation, migration, and differentiation.

Using the TAC software database, we collected information of those miRNAs families, location, length in nucleotides, target gene symbols, and functions. As outlined in Table 3, the 10 selected miRNAs are likely involved in the following signaling pathways: vascular endothelial growth factor and its receptor (VEGF-VEGFR2), mitogen-activated protein kinases (MAPK), phosphatidylinositol 3-kinase-AKT (PI3K-AKT), epithelial growth factor and its receptor (EGR-EGFR), and Transforming growth factor beta (TGF-β). Of these, only three miRNAs (miR-30b-5p, miR-3916, and miR-6893-3p) were subjected to target all these signaling pathways.

### 3.3. Validation of the Expression of miRNA and In Silico Analysis

**Figure 5:**
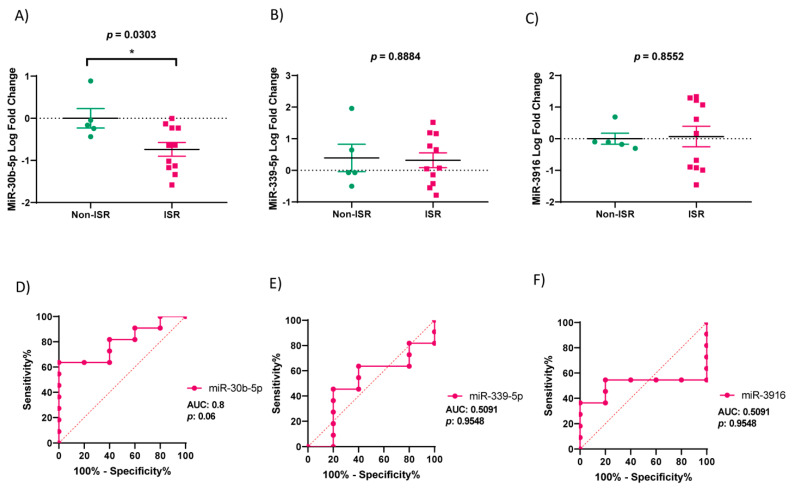
Validation of miRNA expression and ROC analysis in the non-ISR (green) and ISR (rose) patients’ groups. (**A**–**C**) Graphs showing levels of miR-30b-5p, miR-339-5p and miR-3916 in non-ISR and ISR, expressed in log of fold change. Values are means ± S.E.M (n = 16). “*” indicates significance at *p* < 0.05. (**D**–**F**) Graphs illustrate area under the curve (AUC) analysis of ROC indicating sensitivity and specificity of miR-30b-5p, miR-339-5p and miR-3916. Green and rose colors indicate Non-ISR and ISR patients respectively.

Based on the microarrays analyzed, we performed qRT-PCRs to validate the expression of miR-30b-5p, miR-339-5p, and miR-3916. Figure 5A shows that the expression of miR-30b-5p is significantly downregulated in the ISR group, as compared to the non-ISR (*p* = 0.03). However, the expression of miR-339-5p (*p* = 0.89) and miR-3916 (*p* = 0.86) are not significantly different in both groups (Figure 5B,C).

**Figure 6:**
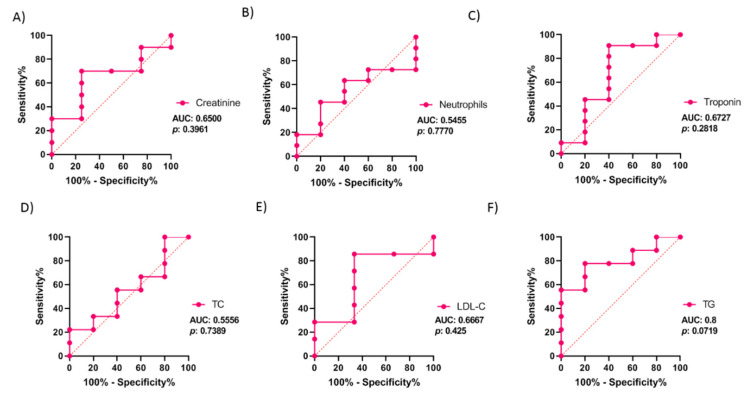
ROC analysis from hemodynamic parameters in patients’ group. (**A**–**F**) Graphs illustrate area under the curve (AUC) analysis of ROC indicating sensitivity and specificity of creatinine (**A**), neutrophils (**B**), troponin (**C**), total cholesterol (TC; **D**), Low-Density Lipoprotein cholesterol (LDL-C; **E**), Triglyceride (TG; **F**). Values are given on the graphs. Rose color indicates ISR patients.

The ROC (receiver operating characteristic) and AUC (area under the curve) were calculated to evaluate the diagnostic value of miR-30b-5p to predict restenosis. As illustrated in Figure 5D–F, the best value of AUC was observed with miR-30b-5p expression, which was 0.8, and tended to be significantly different, with a *p*-value = 0.06 (95% confidence interval (CI): 0.58 to 1.00). Meanwhile, the AUC for miR-339-5p was 0.51 (95% CI: 0.19 to 0.83; *p*-value = 0.95) and was 0.51 for miR-3916 (95% CI: 0.22 to 0.80; *p*-value = 0.96). The analysis indicates that patients with serum levels of miR-30b-5p below −0.54 (log-fold change) could develop restenosis, with a sensitivity and a specificity of 64.6% and 100%, respectively. Moreover, we analyzed the AUC of hemodynamic significant parameters to find out if they could be better diagnostic markers than miR-30b (Figure 6). The hemodynamic parameters did not show any significant *p*-values, whereas TG showed a great AUC (AUC: 0.8, 95%; CI: 0.56 to 1.00; *p*-value: 0.07).

**Figure 7:**
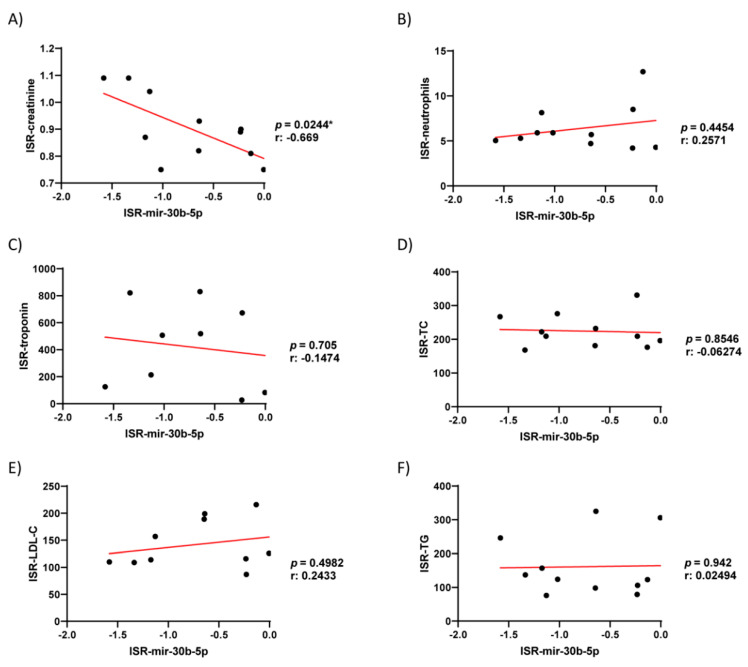
Pearson r Correlation of serum levels of miR-30b-5p respect to different clinical parameters. Linear regression analysis using the concentration/levels of creatinine, (**A**), neutrophils (**B**), troponin (**C**), total cholesterol (TC; **D**), Low-Density Lipoprotein cholesterol (LDL-C; **E**), Triglyceride (TG; **F**); as dependent variable and miR-30b-5p as independent.

Additionally, we studied the possible correlations between the serum levels of miR-30b-5p and hemodynamic clinical parameters, which showed significant differences between the patient groups (creatine, neutrophils, troponin, TC, LDL, and TG) using Pearson’s r correlation. As illustrated in Figure 7, the expression of miR-30b-5p correlated inversely only with creatinine (r: −0.67; *p* = 0.02); in contrast, no significant correlation was observed with the other parameters. Additionally, none of these parameters showed a better AUC value or significance between the patient groups, as compared to miR-30b-5p.

**Figure 8:**
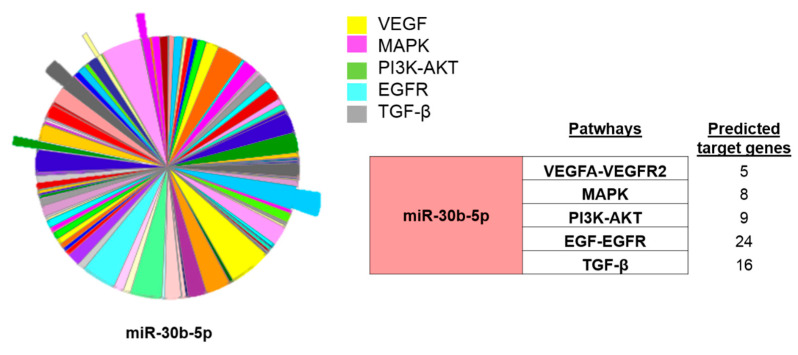
In silico analysis of miR-30-b targets. PANTHER analysis showing the miR-30b-5p predicted target genes involved in different pathways involved in vascular remodeling. The table on the left indicates numbers of miR30b-5p predicted target genes of each signaling pathway. Vascular Endothelial Growth Factor and its Receptor (VEGF-VEGFR), Mitogen-Activated Protein Kinases (MAPK), Phosphatidylinositol 3-Kinase (PI3K-AKT), Endothelial Grow Factor and its receptor (EGR-EGFR) and Transforming Growth Factor (TGF).

Finally, having confirmed the association of miR-30b-5p downregulation with the restenosis incidence, we performed an in-silico analysis using PANTHER software (http://pantherdb.org/ (accessed on 26 March 2021)) to determine the target genes for miR-30b-5p. As highlighted in Figure 8, miR-30b-5p was predicted to target a total of 62 genes related to vascular remodeling and fibrosis, 24 of them associated with EGF/EGFR, 16 with TGF-β, 9 with PI3K-Akt, 8 with MAPK, and 5 with the VEGFA-VEGFR2 signaling pathways. Altogether, these data suggest that miR-30b-5p could have an important role in restenosis and could be a promising biomarker for diagnosis of ISR in patients with angiography recommendation.

## 4. Discussion

CAD is a complex syndrome that is considered a major public health problem, since the epidemiology rates indicate that CAD produces about one-third of all deaths in people older than 35 years [1]. Thanks to major advances using PCI with DES implantation, the mortality caused by CAD has gradually declined over the last decades [11]. Although, according to recent studies, the occurrence of ISR still happen in 5–10% patients with DES and is associated with high mortality and morbidity [12,13]. Therefore, ISR after coronary angioplasty is currently one of the main limitations of PCI therapy with DES implantation, leading to the recurrence of angina pectoris or acute coronary syndromes [14]. Compelling evidence indicates that restenosis happens, after coronary stent implantation, as a result of multifactorial processes involving vascular remodeling due to VSMC proliferation and migration and neointimal hyperplasia, as well as chronic inflammation [15]. Vascular injury during DES implantation [16] might trigger signals promoting the activation of endothelial and smooth muscle cells. Actually, previous data demonstrated that the proliferation and migration of VSMC are directly proportional to the degree of vascular injury [17].

In the present study, we determined that 80% of patients with or without ISR were males who were readmitted to the hospital for a new angiography due to angina pectoris or acute coronary symptoms, similar to what is described in the literature [18]. A serum analysis indicated that classical cardiac markers as creatinine and troponin, but not NT-proBNP or CPK, were increased significantly in patients suffering restenosis, which confirms the severity of the coronary artery obstruction and the corresponding cardiac stress [19,20]. We also observed a significant increase in neutrophil counts in ISR vs. non-ISR patients, indicating inflammation. Similarly, we identified significant differences in the usual lipid parameters such as total cholesterol, LDL-C, and triglycerides in the ISR group, suggesting their implication in atherosclerosis development. Indeed, atherogenesis is associated with high levels of triglycerides expected to deposit within the endothelial and smooth muscle cells [21,22,23]. At the same time, it is well-demonstrated that the association between atherosclerosis and high levels of LDL-C stimulates inflammation and promotes cholesterol accumulation in the blood vessel wall [24]. However, these parameters are not sufficient to distinguish between patients with or without restenosis.

In the last decade, the value of circulating miRNAs as markers in patients with CVDs have been extensively studied [25]. MiRNAs participate in the genetic regulation of hundreds of key proteins involved in different signaling pathways, as those modulating atherosclerosis and cardiovascular remodeling [25,26,27]. Our study revealed significant dysregulation in the levels of miRNAs in ISR as compared to non-ISR patients. A further analysis identified 10 miRNAs with significant fold change differences that are involved in the regulation of genes implicated in VSMC and endothelial cells proliferation, migration, or differentiation. Interestingly, we confirmed that the miR-30b-5p levels were significantly lower in patients with ISR, as compared with non-ISR patients, in agreement with a previous study that showed decreased whole-blood levels of miR-30e-5p in patients with CAD as compared to healthy volunteers [28]. MiR-30b-5p belongs to the miR-30 family composed of miR-30a, miR-30b, miR-30c, miR-30d, and miR-30e. MiR-30 was abundantly expressed in the heart and associated with different CVD [29]. To the best of our knowledge, this study is the first that demonstrated that miR-30b-5p could distinguish between ISR and non-ISR patients. The MiR-30b-5p analysis provided strong AUC and ROC values compared to the miRNA and biochemical parameters, indicating that miR-30b-5p downregulation can distinguish between the patient groups. An in silico analysis indicated that miR-30b-5p was predicted to target genes involved in the VEGF-VEGFR2, MAPK, PI3K-AKT, EGR-EGFR, and TGF-β signaling pathways. The role of these pathways has been extensively investigated in VSMC and endothelial cell proliferation, migration, and differentiation [30,31,32,33,34,35]. In accordance, the expression of miR-30 was decreased in patients with HF, correlating with the increase in VEGF expression [36], while miR-30 downregulation contributes to endoplasmic reticulum stress in cardiac myocyte and VSMCs [37]. Other studies observed a significant decrease in miR-30 family members in medial layers of VSMC, suggesting their role in vascular wall neointima formation induced by a balloon injury of the rat carotid artery [38]. Meanwhile, miR-30b-5p also plays a role in VSMC calcification, a critical step in artery fibrosis, or in atheroma formation [39,40].

## 5. Conclusions

Our study revealed that circulating miR30b-5p could reliably prognose restenosis in patients with implanted DES, which could be potentially helpful in the establishment of an early diagnosis and therapy of ISR.

Study limitations and clinical perspectives:

The present study aimed to validate the proof-of-concept that circulating miRNAs could prognose restenosis occurrence after DES implantation. Nonetheless, in evaluating the results of this study, one must take these limitations into account:

Patients are from one hospital. A large-scale and multicenter study is required to confirm the role of miR-30b-5p as a potential biomarker for ISR.

The small sample size used in this study due to the difficulty of recruiting patients with homogeneous criteria.

The specific role of miR-30b-5p has not been evaluated in terms of the ability to regulate gene expression in human arteries.

A study of the correlation between circulating miR-30b-5p and its expression in coronary arteries might shed light on its real role in vascular remodeling.

Other miRNAs may also have prognostic values in this context, since RNA sequencing or microarray approaches are continuously improving in terms of sensitivity and specificity.

Comparative controlled studies need to be performed in order to determine the best preventive treatment for this entity.

## Figures and Tables

**Table 1:** Data summary (number and percentage) of the patient characteristics. ISR indicates intra-stent restenosis. The other terms are acute coronary symptoms (ACS), coronary artery bypass grafting (CABG), left anterior descending (LAD), left circumflex artery (LCX), right coronary artery (RCA), and percutaneous coronary intervention (PCI). * indicates significant values at *p* < 0.05.

Patients Characteristics	Non-ISR Group (15)	ISR Group (40)	*p*-Value
Age	62 ± 2.73	66.5 ± 1.8	0.1844
Gender, *N* (%)	male 3 (20%)	male 8 (20%)	1
	female 12 (80%)	female 32 (80%)	0.576
Risk Factors, *N* (%)			
Smoking	8 (53.3%)	28 (70%)	0.255
Hypertension	11 (73.3%)	30 (75%)	0.902
Diabetes Mellitus	6 (40%)	25 (62.5%)	0.139
Diabetes Type I	3 (20%)	9 (22.5%)	0.845
Dyslipidemia	12 (80%)	28 (70%)	0.547
Clinical Data, *N* (%)			
Angina Pectoris	10 (66%)	23 (57.5%)	0.34
Acute Coronary Symptoms	3 (20%)	16 (40.0%)	0.382
Chronic Kidney Disease	1 (7%)	0 (0%)	0.103
Heart Failure	1 (7%)	1 (2.5%)	0.471
Stent Details of the 1st PCI			
N° Stenosis	26	70	
Affected Vessels:			
LAD	21 (80.8%)	45 (64.3%)	0.47
LCX	4 (15.4%)	16 (22.8%)	0.09
RCA *	1 (3.8%)	9 (12.9%)	0.04
Stent Details of the 2nd PCI			
N° Stent Implanted		58	
Affected Vessels:			
LAD		31 (53.4%)	
LCX		18 (31.1%)	
RCA		9 (15.5%)	
Treatments			
PCI		36 (90%)	
CABG		3 (7.5%)	
Pharmacologic Treatment		1 (2.5%)	

**Table 2:** Data summary (mean ± S.E.M.) of the hemodynamic parameters from non-ISR and ISR patients evaluated during the second angiography. Creatinine, Creatine phosphokinase (CPK), C-Reactive Protein (CRP), N-terminal natriuretic peptide (NT-proBNP), Total Cholesterol (TC), High-Density Lipoprotein cholesterol (HDL-C), Low-density Lipoprotein Cholesterol (LDL-C), and triglycerides (TG). “*”, “**”, “***” indicate significance at *p* < 0.05, *p* < 0.01 and *p* < 0.001 respectively.

Biochemical Characteristics	Non-ISR Group (15)	ISR Group (40)	*p*-Value
Hemoglobin (g/dL)	125.0 ± 4.1 (84–142)	131.8 (102–180)	n.s
Creatinine (mg/dL)	1.3 ± 0.2 (0.56–2.8)	1.0 ± 0.1 (0.74–1.88)	= 0.05
CPK (ng/mL)	128.3 ± 27.9 (49–327)	187.8 ± 35.6 (48–703)	n.s
CRP (mg/L)	37.9 ± 22.9 (1.4–130.7)	11.7 ± 4.3 (0.5–38.8)	n.s
Troponin (ng/mL) *	44.4 ± 22.3 (5.9–298)	272.1 ± 63.2 (9.3–830)	<0.05
NT-proBNP	5821 ± 5742 (78.2–11563)	6845 ± 2359 (4486–11563)	n.s.
Total Cholesterol (mg/dL) *	170.9 ± 15.1 (76–265.00)	212.5 ± 9.1 (121–331)	<0.05
HDL-C (mg/dL)	50.6 ± 5.5 (19–98)	52.2 ± 3.3 (19–74)	n.s.
LDL-C (mg/dL) ***	92.9 ± 10.2 (45–146)	132.4 ± 8.6 (72.9–216)	<0.01
TG (mg/dl) ***	128.0 ± 13.8 (46–218)	226.0 ± 23.3 (76–446)	<0.001
Erythrocytes (×10e9/L)	4.4 ± 0.1 (3.36–5.3)	4.4 ± 0.1 (3.56–5.82)	n.s.
Leucocytes (×10e9/L)	8.7 ± 0.7 (5.6–15.84)	9.4 ± 0.5 (3.38–15.16)	n.s.
Neutrophils (×10e9/L) **	4.9 ± 0.4 (2.93–7.5)	6.3 ± 0.5 (1.93–12.7)	<0.01

**Table 3:** Selected miRNAs from the array involved in the vascular remodeling pathways. “FC” represented 2 – ΔΔCt, and “Log (FC) > 0” means the gene was upregulated, while “Log (FC) < 0” means the gene was downregulated. FC indicates the fold changes of the microRNA (miRNA) expression in ISR as compared to non-ISR patients. “•” means that the miRNA participates in the corresponding signaling pathway. “-“ means the miRNA does not participate in the signaling pathway. Vascular endothelial growth factor and its receptor R2 (VEGF-VEGFR2), mitogen-activated protein kinases (MAPK), phosphatidylinositol 3-kinase (PI3K-AKT), endothelial grow factor and its receptor (EGR-EGFR), and transforming growth factor beta (TGF-β).

MIRNAS	VEGF-VEGFR2	MAPK	PI3K-AKT	EGF-EGFR	TGF-β	LOG (FC)	*p*-Value
miR-30b-5p	•	•	•	•	•	−1.44	0.0002
miR-3916	•	•	•	•	•	1.36	0.0323
miR-6893-3p	•	•	•	•	•	1.55	0.0040
miR-339-5p	•	•	•	•	-	−1.31	0.0092
miR-449c-5p	•	•	•	-	•	−1.31	0.0047
miR-6737-5p	•	•	•	•	-	−1.35	0.083
miR-365a-5p	•	-	•	•	-	1.33	0.009
miR-518c-3p	-	•	-	•	-	1.31	0.0066
miR-668-5p	-	-	•	-	-	2.26	0.0037
miR-6861-5p	•	-	•	-	-	−1.56	0.0012

## Data Availability

The data presented in this study are available on request from the corresponding author.

## Abbreviations

ACS	Acute Coronary Symptoms
CVD	Cardiovascular Diseases
CRP	C Reactive Protein
CAD	Coronary Artery Disease
CPK	Creatine phosphokinase
DES	Drug-Eluting Stents
EGR-EGFR	Endothelial Grow Factor and its receptor
HF	Heart Failure
HDL-C	High-Density Lipoprotein Cholesterol
LDL-C	Low-Density Lipoprotein Cholesterol
NT-proBNP	N-terminal natriuretic peptide
MAPK	Mitogen-Activated Protein Kinases
PCI	Percutaneous Coronary Intervention
PI3K-AKT	Phosphatidylinositol 3-Kinase
TG	Triglycerides
TC	Total Cholesterol
TGF	Transforming Growth Factor
VEGF-VEGFR2	Vascular Endothelial Growth Factor and its Receptor2
VSMC	Vascular Smooth Muscle Cells
